# The circadian rhythm key gene *ARNTL2*: a novel prognostic biomarker for immunosuppressive tumor microenvironment identification and immunotherapy outcome prediction in human cancers

**DOI:** 10.3389/fimmu.2023.1115809

**Published:** 2023-05-19

**Authors:** Gujie Wu, Hefei Ren, Qin Hu, Huiyun Ma, Hongyu Chen, Lin Zhou, Kun Xu, Liang Ding

**Affiliations:** ^1^ Department of Respiratory medicine, Affiliated Hospital of Nantong University, Nantong, Jiangsu, China; ^2^ Department of Laboratory Medicine, Changzheng Hospital, Naval Medical University, Shanghai, China; ^3^ Department of Chemotherapy, Jiangsu Cancer Hospital, Nanjing Medical University, Nanjing, China

**Keywords:** ARNTL2, prognosis, immunosuppressive tumor microenvironment, immunotherapy, human cancers

## Abstract

**Background:**

Aryl hydrocarbon receptor nuclear translocator-like 2 (ARNTL2) belongs to the b HLH- PAS domain transcription factor family and is one of the key clock genes that control the circadian rhythm. ARNTL2 plays an important role in human biological functions. However, its role in various tumors, especially in the tumor immune microenvironment (TIME) and immunotherapy, remains unclear.

**Methods:**

We integrated data from cancer patients from multiple databases, including the Cancer Genome Atlas, Cancer Cell Lineage Encyclopedia, Genotype Tissue Expression, Human Protein Atlas, cBioPortal, TIMER, and ImmuCellAI, with data from a large clinical study, three immunotherapy cohorts, and *in vitro* experiments to investigate the involvement of ARNTL2 expression in cancer prognosis and immune response.

**Results:**

ARNTL2 displayed abnormal expression within most malignant tumors, and is significantly associated with poorer survival and pathologic staging. Through gene-set enrichment analysis (GSEA) and gene-set variation analysis (GSVA), we found that ARNTL2 not only regulates cell cycle-related functions to promote cell proliferation but also regulates autoimmunity-related functions of the innate and adaptive immune systems, and other immune-related signaling pathways. In addition, ARNTL2 overexpression contributes to an immunosuppressive tumor microenvironment that plays a key role in immunosuppression-related features, such as the expression of immunosuppression-related genes and pathways and the number of immunosuppressive-infiltrating cells, including regulatory T cells (Tregs), tumor-associated macrophages (TAMs), and cancer-associated fibroblasts (CAFs). The group of patients with low ARNTL2 expression who received immune checkpoint inhibitors (ICI) therapy had better response rates and longer survival when compared to those with high ARNTL2 expression.

**Conclusion:**

The findings of this study suggest that ARNTL2 is a potential human oncogene that plays an important role in tumorigenesis and cancer immunity. Elevated ARNTL2 expression indicates an immunosuppressive tumor microenvironment. Targeting ARNTL2 in combination with ICI therapy could bring more significant therapeutic benefits to patients with cancer. Our study sheds light on the remarkable potential of ARNTL2 in tumor immunity and provides a novel perspective for anti-tumor strategies.

## Introduction

Trends in the incidence and mortality of malignant tumors have shown an alarming increase globally over the century; these cancers have thus become a serious threat to the global public health (1). Despite the tremendous advances in cancer treatment in recent years, the prognosis and survival rates of many types of cancer remain unsatisfactory (2, 3). Recent studies have identified disorders of the tumor microenvironment (TME), especially the tumor immune microenvironment (TIME), as one of the main causes of malignant tumor progression (4, 5). Given the great potential of the tumor microenvironment to act as a tumor biomarker and immunotherapeutic target, there is an urgent need for the in-depth study of mechanisms underlying the involvement of the TME in tumor progression.

Aryl Hydrocarbon Receptor Nuclear Translocator Like 2 (ARNTL2) is a clock gene located in the 12p12.2-p11.2 region, which encodes a transcription factor that belongs to the PAS (PER-ARNT-SIM) superfamily and consists of a basic helix-loop-helix (6). PAS superfamily proteins play an essential role in human adaptation to low atmospheric and cellular oxygen levels, environmental pollution, and circadian fluctuations in light and temperature. PAS proteins form transcriptionally active heterodimers with circadian clock proteins and hypoxia-inducible factor (HIF1alpha). ARNTL2 is a paralogous homolog of ARNTL and generates circadian rhythms by regulating its own expression and forming positive and negative feedback pathways (7). Shi et al. (8) showed that ARNTL2 expression can reverse the clock and metabolic phenotypes of ARNTL knockout mice, including rhythmic motor activity and metabolism. At the subcellular and molecular levels, feedback loops of circadian genes control central and peripheral biological clocks. Heterodimers formed by ARNTL or ARNTL2 and CLOCK encode HLH-PAS (helix-loop-helix/PER-ARNT-SIM) transcription factors that bind to E-boxes, which activate PER and CRY transcription. The PER-translated protein becomes extremely unstable after being phosphorylated in the cytoplasm, and it can easily be degraded by ubiquitination, while the CRY-expressed protein can form a complex in the cytoplasm and enter the nucleus to regulate the transcription of the heterodimer encoding HLH-PAS, thus forming a negative feedback pathway (9, 10). When CRY and PER expression decreases, transcriptional repression in turn promotes CLOCK-ARNTL or CLOCK-ARNTL2-mediated restoration of transcriptional function, which in turn generates oscillatory rhythms in circadian gene expression, and there are multiple links between circadian oscillations and the process of tumor formation and progression (11–13). In recent years, it has been successively found that ARNTL2 is highly expressed in a variety of tumors, and is closely related to malignant biological behaviors such as tumor progression, invasion and metastasis (14, 15). However, little is known about whether ARNTL2 is involved in tumor immunity, especially in the regulation of the tumor immune microenvironment and immunotherapy.

In this study, we integrated multi-omics patient data from multiple databases covering 33 cancer types and comprehensively analyzed the involvement of ARNTL2 in these cancer types, including ARNTL2 expression, genetic alteration status, and prognostic role. Our GSEA and GSVA results showed that ARNTL2 is not only involved in tumor progression but is also associated with immune pathways in various tumor types. Finally, we evaluated the correlation between ARNTL2 expression, immune cell infiltration levels, and immune-related gene expression. Elevated ARNTL2 expression predicted the presence of an immunosuppressive tumor microenvironment. Furthermore, analysis of immunotherapy data confirmed that targeting ARNTL2 improves human cancer immunotherapy outcomes. Therefore, this study aims to explore the potential of ARNTL2 in human cancer development, progression, and tumor immunity, providing insights for new anti-tumor strategies.

## Materials and methods

### Data source

The Cancer Genome Atlas (TCGA), Genotype-Tissue Expression (GTEx), and Cancer Cell Line Encyclopedia (CCLE) datasets, along with clinical information and gene expression profiles, were downloaded from the UCSC Xena database (https://xenabrowser.net/datapages/). The Human Protein Atlas (HPA) (http://www.proteinatlas.org/) is a database designed to combine transcriptomics and proteomics to explore the spatial expression levels of transcripts and proteins in different cells, tissues, and organs. It contains the distribution and expression of each protein within 48 human normal tissues, 20 tumor tissues, 47 cell lines, and 12 blood cells.

### Prognostic analysis

The survival information and clinical phenotype data concerning each sample were acquired from TCGA database. A total of four survival prognosis indexes, namely overall survival (OS), disease-specific survival (DSS), progression-free interval (PFI), and disease-free interval (DFI), were used to investigate the correlation of ARNTL2 expression with the prognosis of cancer patients. The R-packages “survival” and “forestplot” were employed to conduct a univariate Cox analysis.

### GSEA and GSVA

Gene Set Enrichment Analysis(GSEA)and Gene Set Variation Analysis(GSVA) were employed to investigate the potential biological process of ARNTL2 in pan-cancer. GSEA was performed using R packages “clusterprofiler”. GSVA is commonly applied for estimating the variation in pathway and biological process activity in the samples of an expression dataset. The Hallmark pathway gene set was downloaded from the Molecular Signatures Database (MSigDB), and the Hallmark pathway scores were obtained for all cancers using the R language “GSVA” package.

### Tumor microenvironment analysis

The R package “ESTIMATE” was used to calculate the stromal score, immune score, and tumor purity score of each patient in TCGA cohort. The association between ARNTL2 expression and these scores were analyzed. The TME-related pathways were obtained and pathway scores were calculated according to the large clinical study (16). We further analyzed the association between ARNTL2 expression and immune cell infiltration, immunomodulatory genes, MHC genes, chemokine/chemokine receptors in pan-cancer level. The visualization of all heatmaps in this step was conducted by the “ggplot2” package. The immune cell infiltration data of TCGA were downloaded from Immune Cell Abundance Identifier (ImmuCellAI) database (http://bioinfo.life.hust.edu.cn/ImmuCellAI#!/) and TIMER2 database (http://timer.cistrome.org/).

### Cell culture

Lung cancer cell lines H1299 were purchased from the American Type Culture Collection (ATCC: Manassas, VA, USA). Cell lines were cultured in RPMI-1640 medium (Gibco, China), with 10% fetal bovine serum (Gibco, China) and were grown in an atmosphere of 5% CO2 at 37°C.

### RNA extraction and qRT-PCR

According to the manufacturer’s protocol, total RNA were isolated using TRIzol reagent (Pufei, Shanghai, China). The primers sequences used for qRT-PCR were obtained from Applied Biosystems (Ribo, Guangzhou, China) as below (5’-3’): ARNTL2 forward - AGGCTTCTTATTTGTGGTTGG, reverse- CAGCGTGGAGATTACTGTGAA. GAPDH forward TGACTTCAACAGCGACACCCA, reverse CACCCTGTTGCTGTAGCCAAA. Relative expression levels were calculated according to the 2-ΔΔCt method. Statistical analyses were performed with GraphPad Prism.

### CCK-8 assay

After transfection with siRNA for 48 h, the cells were transferred to 96-well plates (100 µl cell suspension per well) at a density of 3,000 cells/well in triplicate for each group and incubated in a humidified incubator (Thermo Fisher). CCK-8 reagent (Beyotime, shanghai, China) was added to each well, and the cells were incubated for two additional hours. An iD3 microplate reader was used to measure the absorbance [optical density (OD) value] at 450 nm. The OD value was measured at 0, 24, 48, and 72 hours.

### Scratch wound healing assay

The migration ability of H1299 cells was determined by scratch wound healing assay.H1299cells were harvested and 8 × 105 cells plated in six‐well plates. When the plates yielded cells at about 80% confluence, the monolayer was scraped in a straight line to create a scratch using a 10 μl pipette tip. Debris was then removed using phosphate buffered saline and serum‐free medium was added. Photographs were taken under a microscope at 0,24, and 48 hours.

### Statistical analysis

Mean ± SEM data were compared *via* Student’s t-test in GraphPad Prism 9.3.0. The threshold of significance was considered as P < 0.05.

## Results

### The landscape of ARNTL2 expression

**Figure 1:**
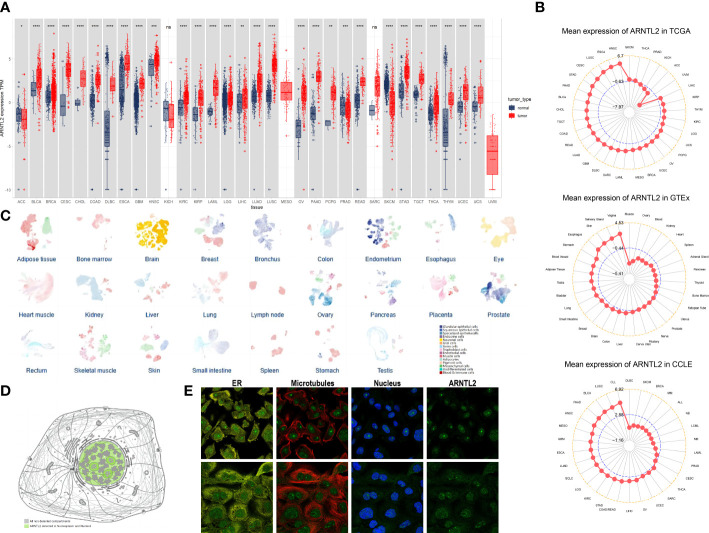
Expression of ARNTL2. **(A)** The expression of ARNTL2 in human cancers. **(B)** ARNTL2 expression in tumor tissues from TCGA cohor, GTEx cohort, and CCLE cohort. **(C)** An overview of all tissues where single cell type expression has been analyzed. **(D)** Intracellular localization of ARNTL2 expression. **(E)** Subcellular localization of ARNTL2 expression within the endoplasmic reticulum (ER), nucleus, and microtubules of the PC-3 and A-431 cells lines. ns, no significance; *p < 0.05, **p < 0.01, ***p < 0.001, and ****p < 0.0001.

To evaluate the expression of ARNTL2 in human cancers, we utilized data from TCGA and GTEx. The analysis indicated that ARNTL2 was overexpressed in 26 out of 33 cancer types, including bladder urothelial carcinoma (BLCA), breast invasive carcinoma (BRCA), cervical squamous cell carcinoma and endocervical adenocarcinoma (CESC), cholangiocarcinoma (CHOL), colon adenocarcinoma (COAD), lymphoid neoplasm diffuse large B-cell lymphoma (DLBC), esophageal carcinoma (ESCA), glioblastoma multiforme (GBM), head and neck squamous cell carcinoma (HNSC), kidney renal clear cell carcinoma (KIRC), kidney renal papillary cell carcinoma (KIRP), acute myeloid leukemia (AML), lower grade glioma (LGG), liver hepatocellular carcinoma (LIHC), lung adenocarcinoma (LUAD), lung squamous cell carcinoma (LUSC), ovarian serous cystadenocarcinoma (OV), pancreatic adenocarcinoma (PAAD), pheochromocytoma and paraganglioma (PCPG), rectum adenocarcinoma (READ), stomach adenocarcinoma (STAD), testicular germ cell tumor (TGCT), thyroid carcinoma (THCA), thymoma (THYM), uterine corpus endometrial carcinoma (UCEC), and uterine carcinosarcoma (UCS). In addition, ARNTL2 was expressed at low levels in only three cancer types, including adrenocortical carcinoma (ACC), prostate adenocarcinoma (PRAD), and skin cutaneous melanoma (SKCM) (**Figure 1A**). After comparing ARNTL2 expression in tumor tissues, we found that ARNTL2 expression was highest in HNSC and lowest in uveal melanoma (UVM) (**Figure 1B**). Among normal tissues, ARNTL2 expression was highest in vaginal tissue and lowest in muscle, while for tumor cell lines, ARNTL2 expression was highest in CLL cell lines using data from the CCLE database. Subsequently, we analyzed the single-cell RNA levels of ARNTL2 in human tissue types and cell type clusters (**Figure 1C**) and detected ARNTL2 in the nucleoplasm and nucleosomes (**Figure 1D**). We verified the intracellular localization of ARNTL2 in the endoplasmic reticulum (ER), microtubules, and nuclei in PC-3 and A-431 cell lines. We observed that ARNTL2 was predominantly located in the nucleus, without overlap with the ER and microtubules (**Figure 1E**).

### Gene alteration of ARNTL2

**Figure 2:**
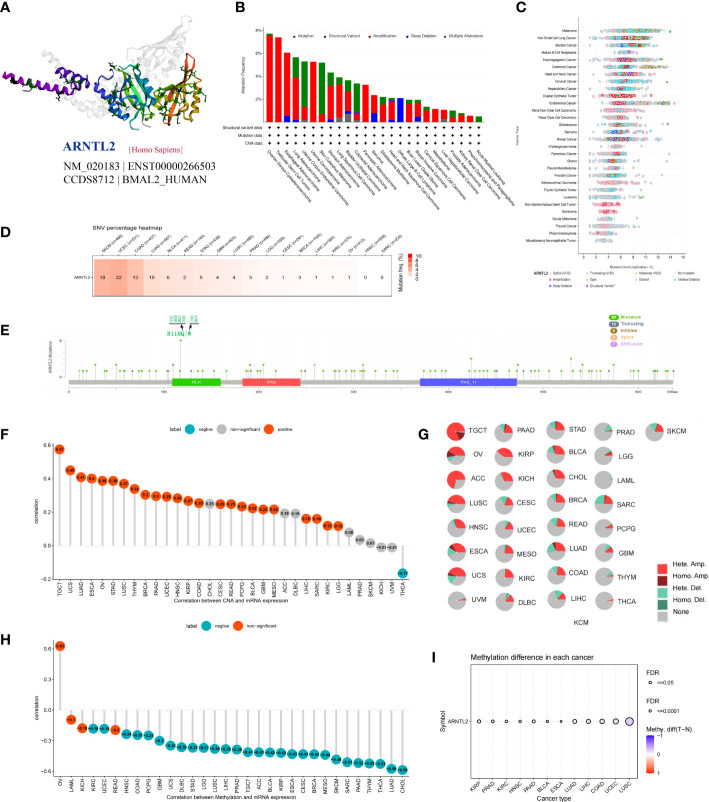
Gene alteration of ARNTL2. **(A)** Three-dimensional structure of ARNTL2. **(B, C)** Genetic alteration frequency and counts of ARNTL2. **(D, E)** Deleterious mutation percentage and mutation sites of the ARNTL2. **(F)** Correlation between ARNTL2 expression and CNV. **(G)** Pie plot summarizing the CNVs of ARNTL2 in the indicated tumor types. **(H)** The correlation between ARNTL2 expression and DNA methylation. **(I)** DNA methylation difference in each cancer.

Gene mutations, copy number alterations (CNAs), and DNA methylation are closely associated with tumor development and progression. Using the cBioPortal platform, we conducted an overall analysis of ARNTL2 mutations in the tumor tissues of 10,953 patients. After exploring the three-dimensional structure of ARNTL2, it was observed that ARNTL2 amplification accounted for the largest proportion among all mutation types, with the highest amplification being observed in ovarian serous cystadenocarcinoma (7.53%), followed by TGCT (7.38%) and cervical squamous cell carcinoma (5.49%). UCEC, SKCM, and colorectal adenocarcinoma had a substantial number of mutations (**Figures 2A, B**). **Figure 2C** illustrates the mutation counts of ARNTL2. For the single-nucleotide variant (SNV) percentage, the total deleterious mutation percentage (i.e., the number of samples with at least one deleterious mutation site/the number of samples with SNV mutation data) showed the highest deleterious mutation frequency of ARNTL2 in UCEC, SKCM, and COAD (**Figure 2D**). Among the three most common mutation sites, HLH, PAS, and PAS-11, mutations in HLH were associated with UCEC, SKCM, BRCA, COAD, and STAD progression (**Figure 2E**). Upon analyzing the relationship between ARNTL2 expression and CNV, we found that ARNTL2 expression had a significant positive correlation with CNV in all cancer types assessed (**Figure 2F**). We also analyzed the CNV of ARNTL2. **Figure 2G** shows a substantial presence of CNVs in ARNTL2, including heterozygous amplification, heterozygous deletion, homozygous amplification, and homozygous deletion, in the 33 types of cancer assessed. DNA methylation, as an epigenetic modification, usually leads to the disruption of immune cell homeostasis and tumor immunosurveillance functions, which results in cancer development and proliferation. Therefore, we further calculated the correlation levels between ARNTL2 and promoter methylation. ARNTL2 expression was significantly correlated with the methylation status in 28 tumors, with the highest negative correlations in CHOL and LUAD (**Figure 2H**). **Figure 2I** shows the methylation difference in cancers. These results suggest a potential function of ARNTL2 in tumorigenesis and progression of most human cancers.

### Prognostic role of ARNTL2

**Figure 3:**
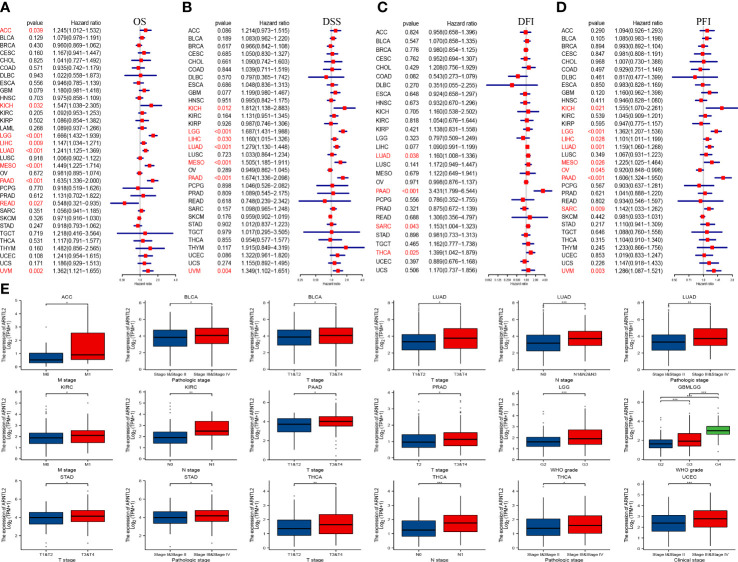
Prognostic value of ARNTL2. **(A)**The univariate Cox regression OS analysis of ARNTL2. Red color represents significant results (p < 0.05). **(B-D)** The univariate Cox regression analysis of DSS **(B)**, DFI **(C)**, and PFI **(D)**. **(E)** Differential expression of ARNTL2 in pathological stages in specified tumour types. *P < 0.05, **P < 0.01, ***P < 0.001.

To investigate the correlation between ARNTL2 expression and cancer prognosis, we analyzed the hazard ratio statistics of overall survival (OS), disease-specific survival (DSS), disease-free interval (DFI), and progression-free interval (PFI) for each cancer type included in this study. Based on univariate Cox regression analysis, ARNTL2 was identified as a significant risk factor for OS in ACC, KICH, LGG, LIHC, LUAD, MESO, PAAD, READ, and UVM (**Figure 3A**). Moreover, Cox regression analysis of the DSS supported ARNTL2 as a risk factor for KICH, LGG, LIHC, LUAD, MESO, PAAD, and UVM (**Figure 3B**). For DFI, high ARNTL2 expression predicted a shorter DFI in patients with LUAD, PAAD, SARC, and THCA (**Figure 3C**). Additionally, Cox regression analysis of the PFI revealed that ARNTL2 is a detrimental factor for patients with KICH, LGG, LIHC, LUAD, MESO, PAAD, SARC, and UVM (**Figure 3D**). Finally, we examined the correlation between ARNTL2 expression and various pathological cancer stages and found that upregulation of ARNTL2 was associated with advanced stages of various cancers (**Figure 3E**). These findings suggest that ARNTL2 plays an important role in the prognosis of patients with cancer.

### Protein-protein interaction network

**Figure 4:**
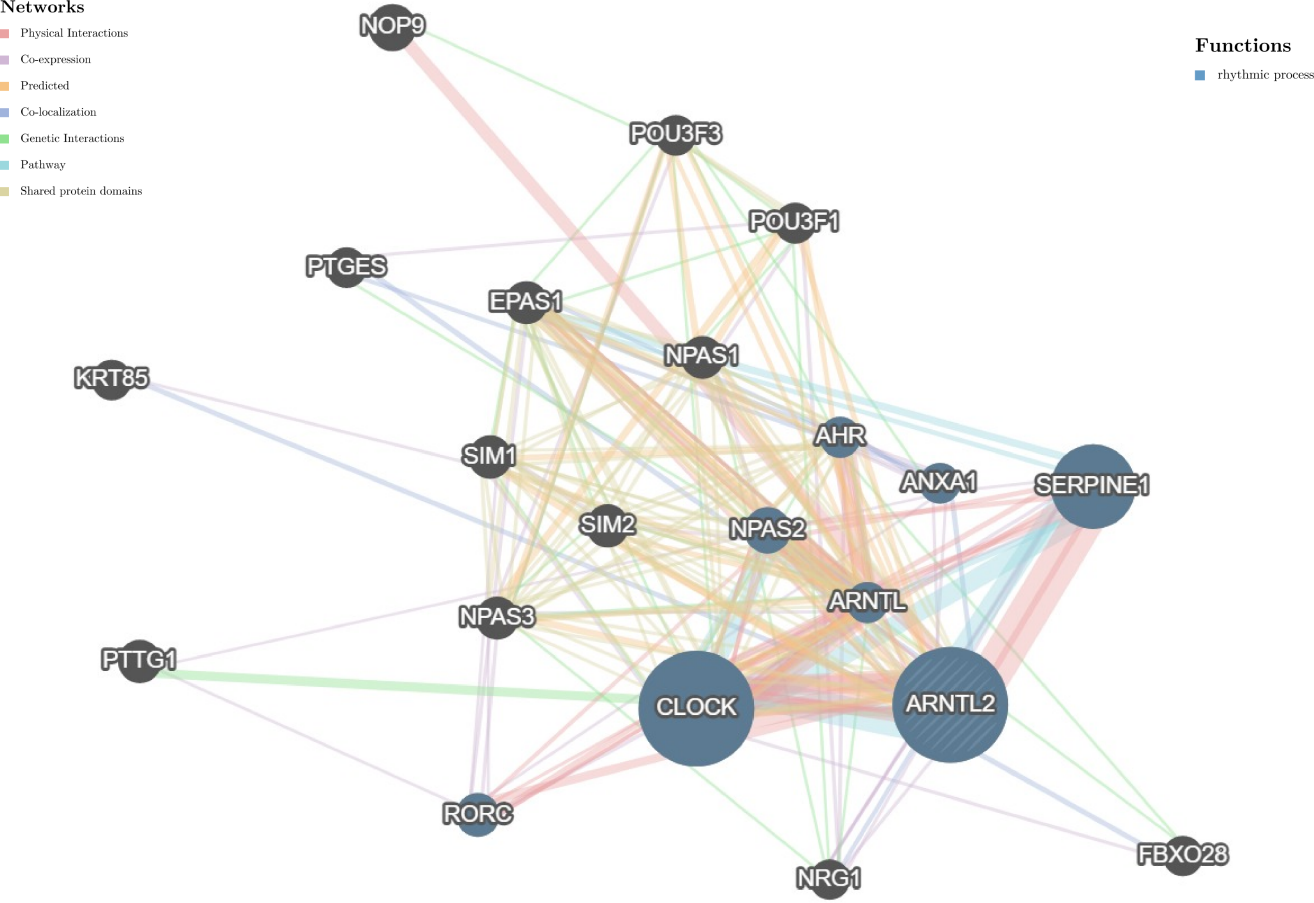
Protein-protein interaction network for ARNTL2. Correlation between ARNTL2 and 20 functionally similar genes. The color of the nodes correlates with protein function, while the color of the lines represents the type of protein interactions.

Circadian rhythm disorders have been recognized as potential independent risk factors for the development of cancer (16). Considering the important role of ARNTL2 in adapting to circadian fluctuations, we constructed a protein-protein interaction network of ARNTL2 using the GeneMANIA database to analyze its potential functions in cancer development and progression in conjunction with other synergistic genes. As shown in **Figure 4**, the hub node representing ARNTL2 is surrounded by nodes of 20 genes significantly associated with it. Among these 20 genes, we found that ARNTL2, CLOCK, SERPINE1, ARNTL, NPAS2, ANXA1, RORC, and AHR are mutually involved in the regulation of rhythmic processes. The clock gene CLOCK showed the strongest correlation with ARNTL2, followed by the hypoxia-inducible gene SERPINE1. This further underscores the potential involvement of ARNTL2 in regulating circadian rhythms and hypoxia, and its potential role in cancer development and progression.

### Biological function

**Figure 5:**
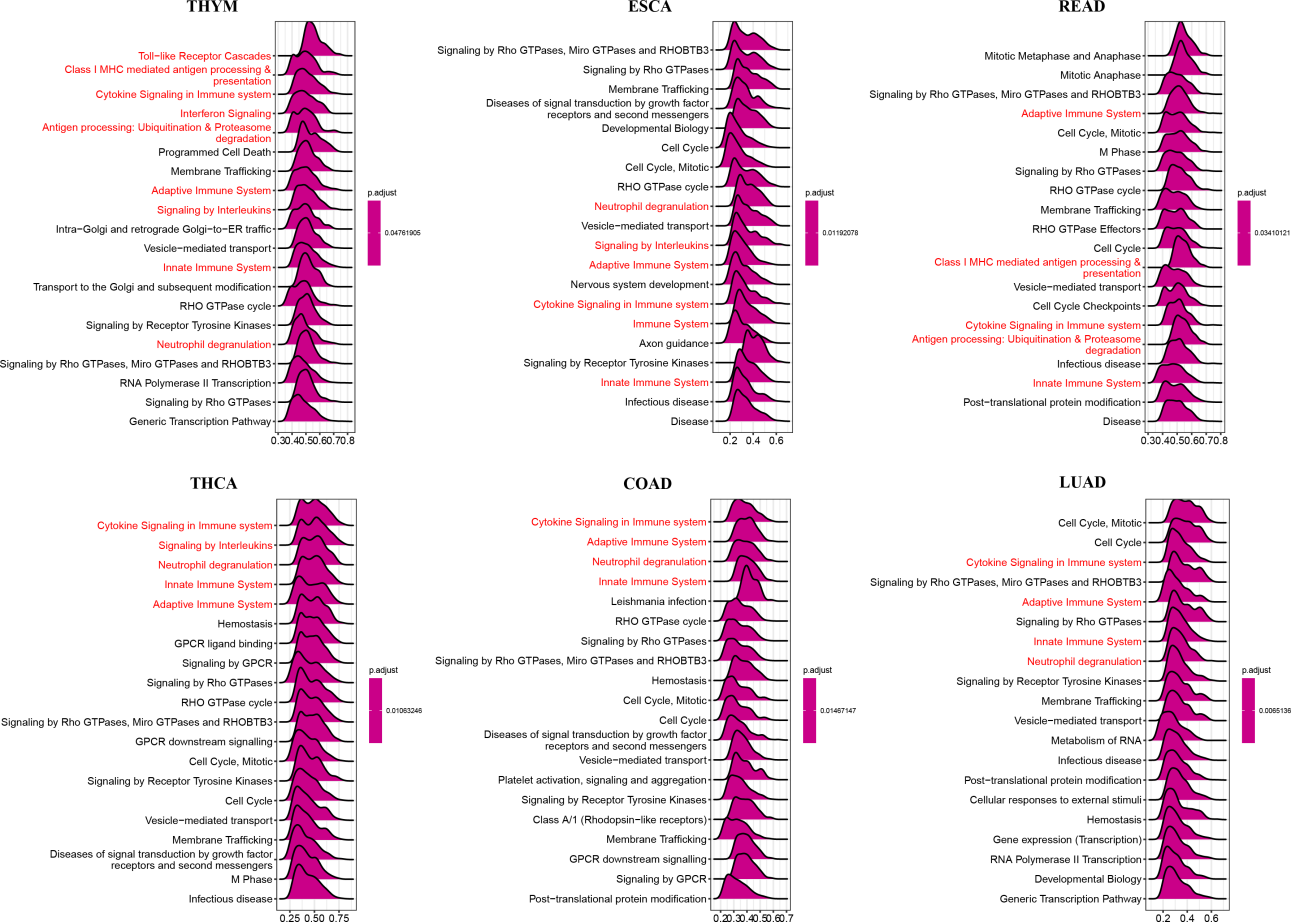
GSEA of ARNTL2. The top 20 significant pathways of ARNTL2 GSEA results across the indicated tumor types. Red color represents immune-related pathways.

To investigate the biological processes related to ARNTL2 in human cancers, we conducted a GSEA algorithm analysis using the “clusterprofiler” tool and identified 18 tumors that yielded comparable findings (**Figure 5** and **Supplementary Figure 1**). We found that ARNTL2 is associated with cycle-related pathways in human cancers, such as ‘Cell Cycle,’ ‘Signaling by Rho GTPases, Miro GTPases and RHOBTB3,’ and ‘Vesicle-mediated transport.’ In addition, ARNTL2 was extensively involved in immunomodulation-related pathways, particularly those related to adaptive immunity, innate immunity, and cytokine signaling in the immune system. Based on these results, it is apparent that ARNTL2 influences both the growth of tumors and the immune system’s response to them.

**Figure 6:**
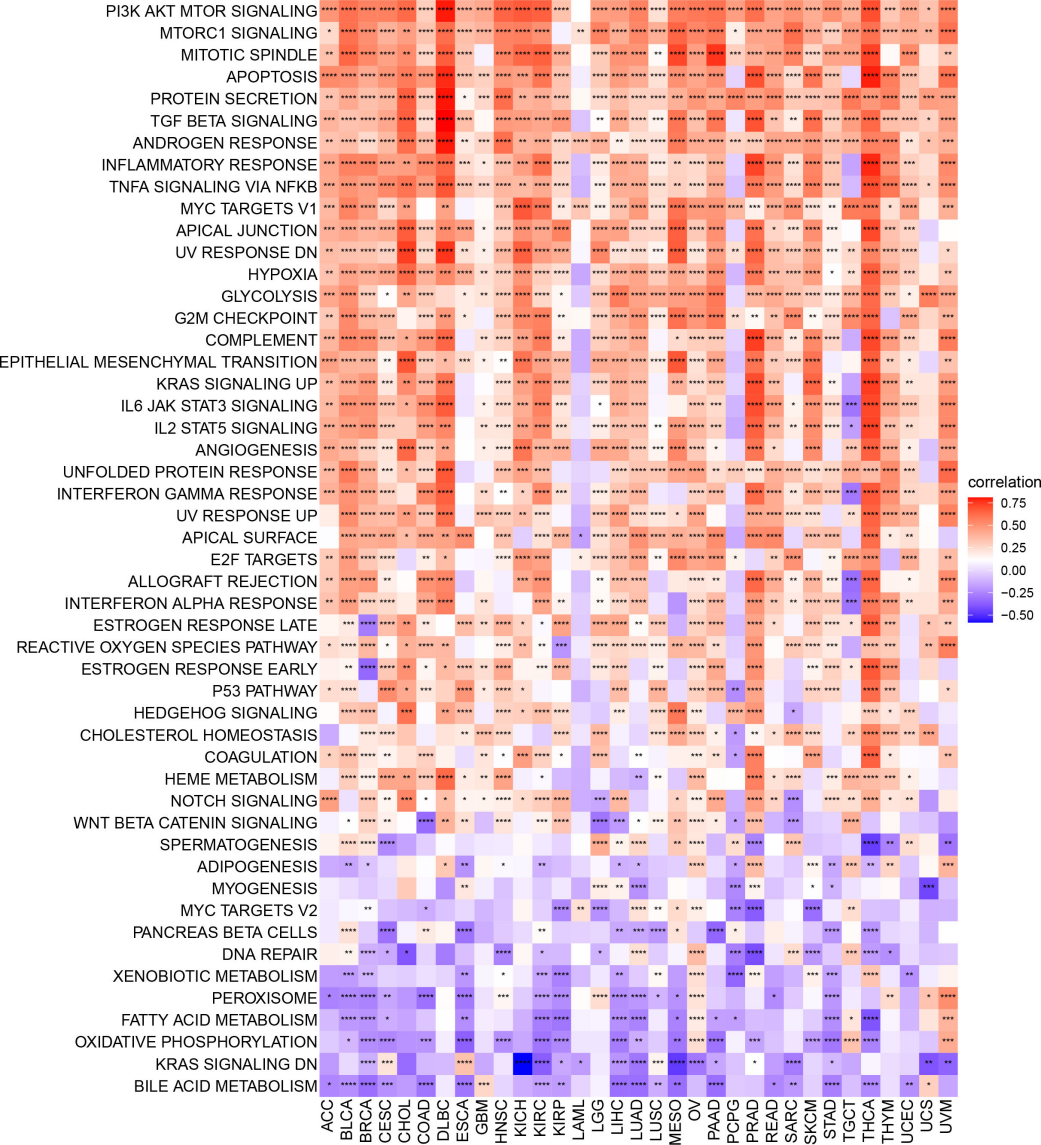
GSVA of ARNTL2. GSVA results of 50 hallmark pathways from the MSigDB. *P < 0.05, **P < 0.01, ***P < 0.001, ****P < 0.0001.

To identify signaling pathways that may be affected by ARNTL2 expression, we performed GSVA scoring based on 50 HALLMARK pathways (**Figure 6**). We observed that ARNTL2 expression was positively correlated with multiple cancer-promoting pathways, including the hypoxia signaling pathway, KRAS signaling pathway, P53 signaling pathway, and MYC signaling pathway. ARNTL2 expression also appeared to be extensively involved in immune-related signaling pathways, such as the interferon-alpha response, interferon-gamma response, TGF-Beta signaling pathway, IL-2/STAT5 signaling pathway, and IL6/JAK/STAT3 signaling pathway. Each of these pathways was also shown to have a strong association with TIME.

**Figure 7:**
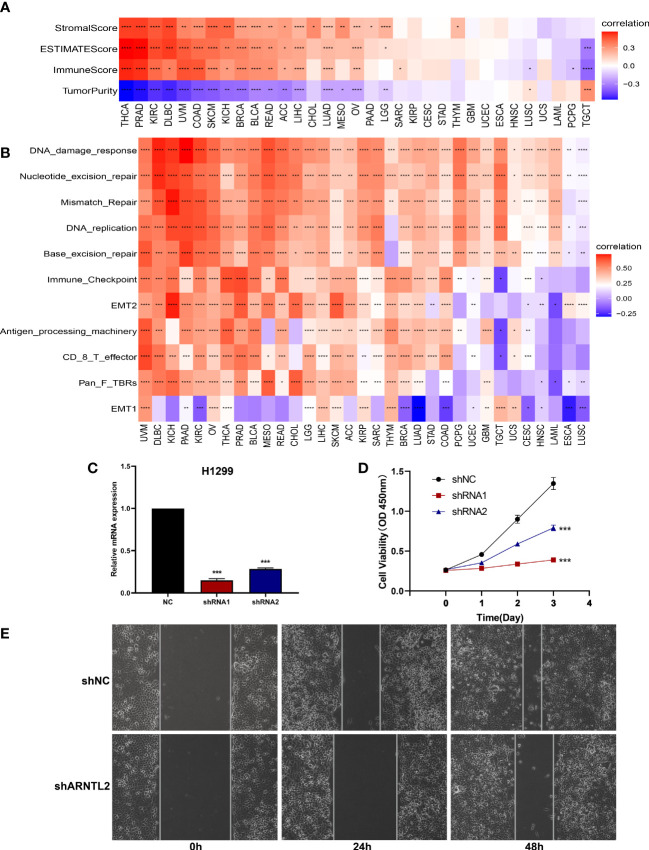
Function and verification of ARNTL2. **(A)** Heatmap represents the correlation between ARNTL2 expression and TME scores in human cancers. **(B)** The TME-related pathway involved in ARNTL2 was identified by analyzing data from a largescale study. **(C)** After H1299 cells were transfected with si-ARNTL2, the level of ARNTL2 was evaluated by qRT-PCR. **(D)** The cell viability of cells was examined by CCK-8 assay. **(E)** The cell invasion of H1299 cells was examined by scratch assay. *P < 0.05, **P < 0.01, ***P < 0.001, ****P < 0.0001.

To understand the role of ARNTL2 in TIME in human cancers, we assessed the association between ARNTL2 expression and stromal and immune scores using the “ESTIMATE” algorithm (**Figure 7A**). The results revealed that ARNTL2 expression was capable of influencing immune scores, stromal scores, and ESTIMATE scores in most tumors. To validate this finding, we obtained immune data and calculated the TME-related pathways involved in ARNTL2, including immune-related pathways, tumor-related pathways, and DNA repair-related pathways, based on a large clinical study. This revealed the same outcome that ARNTL2 was closely associated with tumor proliferation and immune-related pathways (**Figure 7B**). To further support the proliferation-promoting role of ARNTL2, we successfully knocked down the expression of ARNTL2 in H1299 cells using two ARNTL2 shRNAs (**Figure 7C**). We then performed CCK8 assays with these cells and found that ARNTL2 knockdown significantly reduced the viability of H1299 lung cancer cells (**Figure 7D**). As detailed in **Figure 7E**, the migratory activity of tumor cells was also inhibited after 24 and 48 hours when ARNTL2 expression was knocked down by shRNA1 in the H1299 cell line. This finding is consistent with our hypothesis that ARNTL2 is oncogenic. Combined with our GSEA and GSVA results, these findings confirm that ARNTL2 has a broad pro-oncogenic effect. Then, we further explored the mechanism of action of ARNTL2 in TIME.

### The relationship between ARNTL2 and immunosuppressive tumor microenvironment

**Figure 8:**
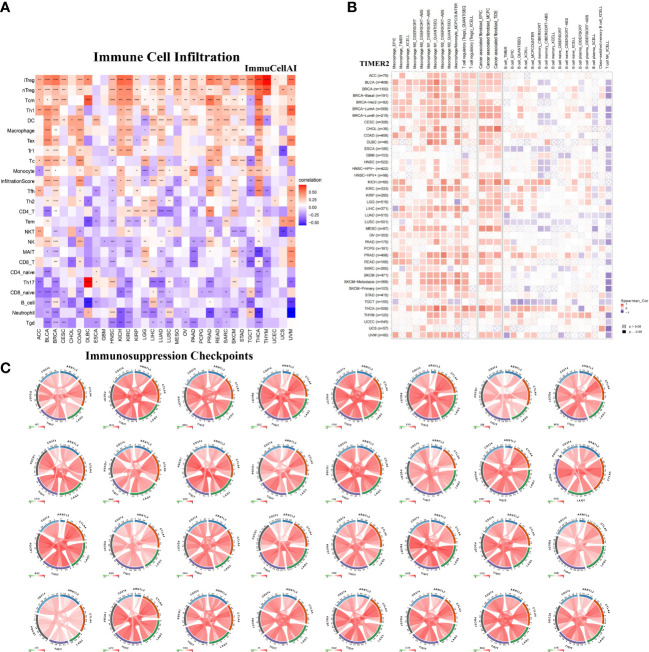
Relationship between ARNTL2 expression and immunosuppressive tumor microenvironment. **(A)** Correlation between ARNTL2 expression and different immune cells from ImmuCellAI database. **(B)** Correlation between ARNTL2 expression and different immune cells from TIMER2 database. **(C)** Correlation of ARNTL2 expression with CD274 (PD-L1), PDCD1 (PD-1), CTLA4, LAG3 and TIGIT in human cencers. Red represents positive correlation, blue represents negative correlation, and the darker the color, the stronger the correlation. *P < 0.05, **P < 0.01, ***P < 0.001, ****P < 0.0001.

To further examine the relationship between ARNTL2 and the tumor immunosuppressive microenvironment, we performed a correlation analysis of ARNTL2 expression, immune cell infiltration, and immune-related genes. First, we analyzed the relationship between ARNTL2 expression and immune cell infiltration using immune data from ImmuCellAI and TIMER2 databases, respectively (**Figures 8A, B**). The escape of immune surveillance by tumor cells may be caused by the dysregulation of infiltrating immune cells that occurs throughout the onset and progression of tumors. Results from both databases showed that in most malignancies, ARNTL2 expression positively correlated with the infiltration of immunosuppressive-related cells, including regulatory T cells (Tregs) and tumor-associated macrophages (TAMs), but suppressed the infiltration of immune-promoting-related cells such as B cells, NK/NKT cells, CD4+ T cells, and CD8+ T cells. In addition, cancer-associated fibroblasts (CAFs) were also found to play an essential role in the formation of ARNTL2-mediated tumor immunosuppressive microenvironment. These results revealed that a high level of ARNTL2 expression may contribute to an immunosuppressive tumor microenvironment in patients with cancer.

**Figure 9:**
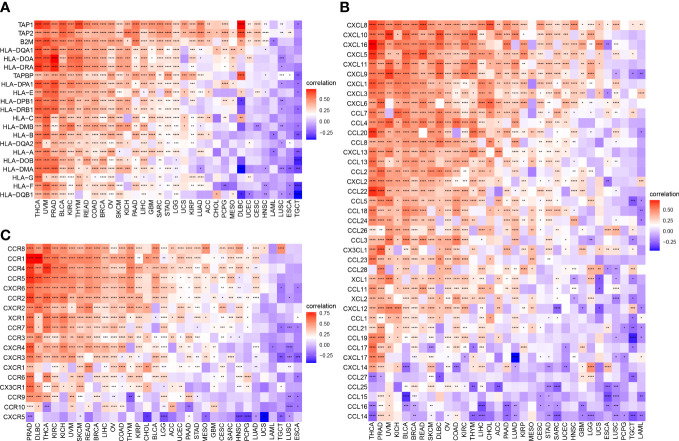
Relationship between ARNTL2 expression and immune-related genes. **(A, B)** MHC genes. **(B)** Chemokines. **(C)** Chemokine receptors. Red represents positive correlation, blue represents negative correlation, and the darker the color, the stronger the correlation. *P < 0.05, **P < 0.01, ***P < 0.001, ****P < 0.0001.

To better understand the association between ARNTL2 and the immunosuppressive tumor microenvironment, we examined the degree to which immune-related genes and ARNTL2 were correlated with one another. The expression of ARNTL2 was shown to have a positive association with the majority of immunosuppression checkpoints in most malignancies assessed, including TIGIT, PDCD1 (PD-1), LAG3, CTLA4, and CD274 (PD-L1) (**Figure 8C**). Additionally, we provided further evidence supporting ARNTL2 as strongly associated with immunoregulatory genes, including MHC genes (**Figure 9A**), chemokines (**Figure 9B**), and chemokine receptors (**Figure 9C**). Genes associated with the immune system, including CCR2, CCL2, CXCR4, and CCR5, are among those that participate in the recruitment of TAMs during the initiation and progression of tumors. TAMs release immune-associated genes, including CCL3, CCL4, CCL5, and CCL22, which enable them to mediate the immune suppression activities of T cells. These findings support ARNTL2 as a key regulatory factor in an immunosuppressive tumor.

### The relationship of ARNTL2 with immunotherapy response

**Figure 10:**
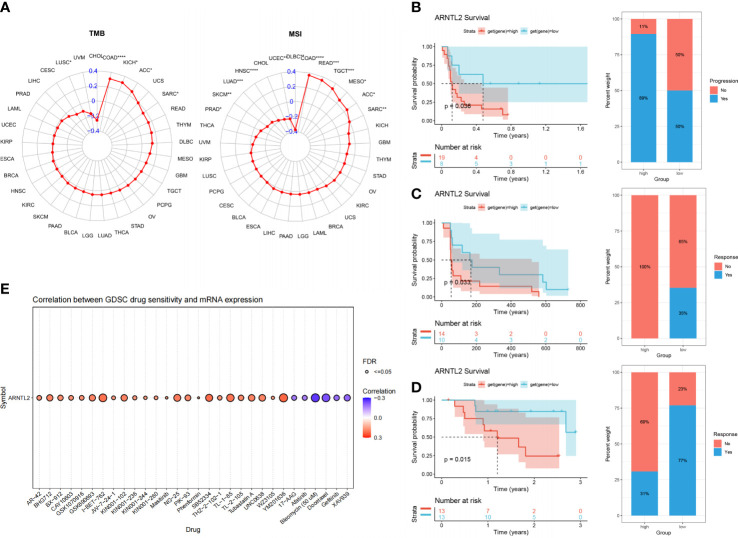
The association between ARNTL2 expression and immunotherapy response. **(A)** Radar plot of the correlation between ARNTL2 expression and TMB and MSI. **(B)** The Kaplan-Meier PFS analysis and percentage of responsive patients in high- and low-ARNTL2 expression groups of GSE135222 cohort. **(C)** The Kaplan-Meier PFS analysis and percentage of responsive patients in high- and low-ARNTL2 expression groups of GSE91061 cohort. **(D)** The Kaplan-Meier OS analysis and percentage of responsive patients in high- and low-ARNTL2 expression groups of GSE78220 cohort. **(E)** The correlation between gene expression and the sensitivity of GDSC drugs (top 30) in human cancer. *P < 0.05, **P < 0.01, ***P < 0.001, ****P < 0.0001.

With the continuous improvement of immune checkpoints in recent years, immune checkpoint inhibitors (ICIs) have become increasingly used in tumor immunotherapy and have played an influential role in clinical practice. TMB and MSI scores have high predictive value for the response to ICI therapy and prognosis (17). We found that ARNTL2 expression was correlated with TMB in five cancer types and MSI in twelve cancer types (**Figure 10A**). Based on these findings, we hypothesized that ARNTL2 expression may affect the patients’ response to immunotherapy. To test this hypothesis, we gathered immunotherapy data from three cohorts and estimated ARNTL2 expression in various malignancies. Through Kaplan-Meier analysis, we found that low ARNTL2 expression was correlated with longer overall survival and a better overall response rate in patients receiving ICI treatment (**Figures 10B–D**). These findings suggested that ARNTL2 expression has an important impact on patient response to immunotherapy. Finally, we collected the IC50 values of 265 small molecule drugs and their corresponding mRNA gene expression from the Cancer Drug Sensitivity Genomics (GDSC) database in 860 cell lines to identify ARNTL2-related antitumor drugs (**Supplementary Table 1**). **Figure 10E** shows the top 30 antitumor drugs with the highest relevance, among which I-BET-762 small molecule inhibitors that inhibit the function of BET (bromodomain and extra-terminal) family proteins were able to inhibit several genes related to carcinogenesis in tumor cells and immune cells, including c-Myc, pSTAT3, and pERK (18). This not only leads to the arrest of tumor cell proliferation but also alters the distribution of immune cell populations in the tumor microenvironment. In mouse models of lung cancer, I-BET-762 significantly delayed tumor development. These drug options also provide additional choices for the precision treatment of lung adenocarcinoma patients.

## Discussion

The invasion and metastasis of malignant tumors is a complex and multifactorial process. However, recent studies have shown that the tumor microenvironment (TME) plays a crucial role in the malignant evolution of tumors. The TME refers to the environment surrounding tumor cells, which is composed of immune cells, fibroblasts, endothelial cells, and neuronal cells, as well as their extracellular matrix (ECM) proteins, signaling molecules, and surrounding blood vessels during tumor growth. This environment is essential for maintaining tumor cell proliferation, differentiation, metabolism, and functional activities, and has become one of the hallmark features of tumors (19). The TME is characterized by three major categories: hypoxia, chronic inflammation, and immunosuppression. During tumor development, the TME interacts with tumor cells and jointly mediates immune tolerance of the tumor, which affects the clinical effect of immunotherapy. Tumor cells and TME are complementary, and the interaction between the two is crucial for the development and progression of tumors. Therefore, a full understanding of the TME will facilitate the further development of effective therapies for human cancer.

In this study, we conducted an evaluation of the expression and prognostic significance of ARNTL2 in human cancers. Our results revealed that ARNTL2 is highly expressed in 26 different cancer types, including BLCA, BRCA, CESC, CHOL, COAD, DLBC, ESCA, GBM, HNSC, KIRC, KIRP, LAML, LGG, LIHC, LUAD, LUSC, OV, PAAD, PCPG, READ, STAD, TGCT, THCA, THYM, UCEC, and UCS. Further assessment of the prognostic role of ARNTL2 indicated that upregulation of this gene is significantly associated with unfavorable pathological staging and poor prognosis in several different cancer types. Notably, we also found that ARNTL2 expression is influenced by gene mutations, copy number alterations (CNA), and DNA methylation. Our findings suggest that ARNTL2 could serve as a valuable biomarker for predicting the prognosis of tumor patients, potentially aiding in the development of personalized treatment strategies. In recent years, circadian rhythm disorders have emerged as an independent risk factor for cancer (20). As a key gene in circadian rhythm, ARNTL2 has been reported to promote the progression of pancreatic ductal adenocarcinoma through the TGF/BETA pathway (14). Another study showed that deletion of ARNTL2 could inhibit colon carcinogenesis through inactivation of the PI3K/AKT pathway (15). Therefore, it is essential to investigate and clarify the potential relationship between malfunctional genes and tumor development for the prevention and treatment of tumors. Interestingly, we observed that ARNTL2 not only promotes multiple cancer-promoting pathways, including the hypoxia signaling pathway, KRAS signaling pathway, P53 signaling pathway, and MYC signaling pathway, but it is also extensively involved in immune-related signaling pathways such as the interferon-alpha response, interferon-gamma response, TGF-Beta signaling pathway, IL-2/STAT5 signaling pathway, and IL6/JAK/STAT3 signaling pathway. The circadian clock influences various aspects of tumor biology, including cell proliferation, DNA damage response, and drug sensitivity. In addition, the hypoxic microenvironment is a common feature of solid tumors and is known to play a crucial role in tumor progression and therapy resistance. Recent studies have suggested that the circadian clock can regulate the hypoxia response pathway and that the interaction between circadian rhythm and hypoxia may contribute to tumor growth and metastasis (21). We confirmed the role of ARNTL2 in tumor progression through large clinical data and *in vitro* experiments. Subsequently, considering the tremendous potential of ARNTL2 in cancer immunology, we further explored the mechanism of action of ARNTL2 in TME.

The dysfunction of TME, particularly TIME, is a well-established determinant of tumorigenesis and progression (15–17). The TIME is now being extensively explored as a therapeutic target for cancer, as it has been shown to facilitate tumor growth and is associated with poor responses to immunotherapy (19). Immune cells play a crucial role in the TIME and are involved in a wide range of immunological actions and responses (21, 22). Tumor-associated macrophages (TAMs) are the most abundant immune cells in the TME and can be divided into two categories based on their phenotypic characteristics and local microenvironment: pro-inflammatory “classically activated macrophages (CAM)” and anti-inflammatory “alternatively activated macrophages (AAM)”. CAMs perform immunosurveillance and antigen presentation functions, secrete pro-inflammatory cytokines and chemokines, and participate in positive immune responses (23). In contrast, AAMs have less antigen-presenting capacity and play an important role in immune regulation by secreting suppressive cytokines such as IL-10 and/or TGF-β, which downregulate antitumor immune responses (24). In general, TAMs are characterized by polarization toward AAM-type macrophages in the TME (25). After being “trained” as AAMs, TAMs promote tumor cell survival, tumor progression, and metastasis through various signaling pathways (26, 27). Additionally, the accumulation of regulatory T cells (Tregs) in the TME facilitates malignant cells to evade killing by cytotoxic CD8+ T cells and reduces the anti-tumor immune response, which is thought to be an important driver of tumor immune evasion (27). Tumor cells can also directly or indirectly remodel TAMs and cancer-associated fibroblasts (CAFs), thereby inhibiting the cytotoxicity of anti-tumor immune cells and subsequently exerting immunosuppressive and tumor-promoting effects (28–31). To further explore the connection between ARNTL2 and the immunosuppressive milieu, we conducted a correlation analysis between immune-related genes and ARNTL2. Our study found significant correlations of ARNTL2 with most immunosuppressive checkpoints, including PD-1, PD-L1, CTLA4, LAG3, and TIGIT. Moreover, TAM recruitment depends on immune-related genes such as CCL2, CCR2, CXCR4, and CCR5, which can promote cancer development by promoting immunosuppression (32, 33). Activating Tregs can increase the expression of numerous immune checkpoints that suppress the immune system, including PD-1, CTLA-4, TIM-3, and TIGIT. They can also increase the expression of numerous molecules and transporters that contribute to T cell dysfunction, including CD39, CD73, and CCR4 (34, 35). These results reaffirmed the immunosuppressive role of ARNTL2 in human cancers and indicated that ARNTL2 overexpression contributes to an immunosuppressive tumor microenvironment.

## In conclusion

Our findings suggest that ARNTL2 could be a promising prognostic biomarker and therapeutic target for human cancers. High expression of ARNTL2 may contribute to the development of an immunosuppressive tumor microenvironment. Targeting ARNTL2 in combination with ICI therapy could bring significant therapeutic benefits to patients with cancer. Our study explores for the first time the remarkable potential of ARNTL2 in tumor immunity and provides a new perspective for anti-tumor strategies.

## Data availability statement

The original contributions presented in the study are included in the article/**Supplementary Material.** Further inquiries can be directed to the corresponding author.

## Ethics statement

TCGA belong to public databases. The patients involved in the database have obtained ethical approval. Users can download relevant data for free for research and publish relevant articles. Our study is based on open source data, so there are no ethical issues and other conflicts of interest. The Ethics Committee of local legislation and institutional has granted exemptions from approval for research related to the use of such public databases.

## Author contributions

GW, QH, and HR acquired the data and drafted the manuscript. HM and HC provided statistical analysis and technical support. LZ and KX conceived the idea and designed the study. LD made significant revisions to the manuscript and ultimately approved it for publication. All authors contributed to the article and approved the submitted version.

## Supplementary material

Supplementary Figure 1 GSEA of ARNTL2 in other 12 tumor types.

Supplementary Table 1 The correlation between gene expression and the sensitivity of GDSC drugs.
