# Supplementary figures and images for: The circadian rhythm key gene ARNTL2: a novel prognostic biomarker for immunosuppressive tumor microenvironment identification and immunotherapy outcome prediction in human cancers
Source: Front Immunol. 2023 May 19;14:1115809. doi: 10.3389/fimmu.2023.1115809 (PMC10237319; doi:10.3389/fimmu.2023.1115809)

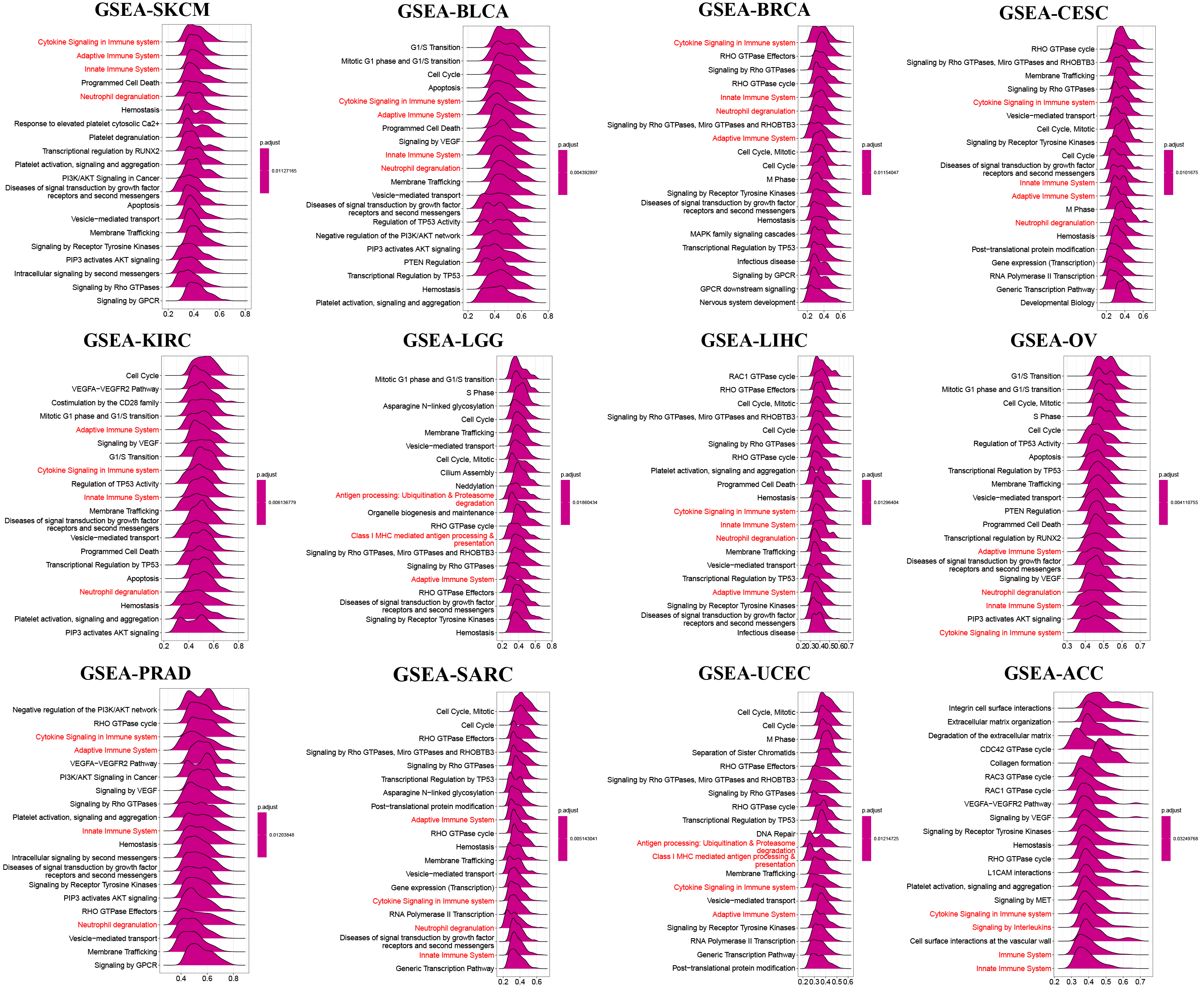

Supplement: Supplementary Figure 1 — GSEA of ARNTL2 in other 12 tumor types. [file Image_1.tif]
